# Metabolomics of Papanicolaou Tests for the Discovery of Ovarian Cancer Biomarkers

**DOI:** 10.3390/metabo14110600

**Published:** 2024-11-07

**Authors:** Samyukta Sah, Elisabeth M. Schwiebert, Samuel G. Moore, Ying Liu, David A. Gaul, Kristin L. M. Boylan, Amy P. N. Skubitz, Facundo M. Fernández

**Affiliations:** 1 School of Chemistry and Biochemistry, Georgia Institute of Technology, Atlanta, GA 30322, USA; ssah9@gatech.edu (S.S.); eschwiebert3@gatech.edu (E.M.S.); david.gaul@chemistry.gatech.edu (D.A.G.); 2 Petit Institute of Bioengineering and Bioscience, Georgia Institute of Technology, Atlanta, GA 30322, USAyl80@gatech.edu (Y.L.); 3 Department of Laboratory Medicine and Pathology, University of Minnesota, Minneapolis, MN 55455, USA; boyla002@umn.edu (K.L.M.B.); skubi002@umn.edu (A.P.N.S.)

**Keywords:** ovarian cancer (OC), Papanicolaou (Pap) tests, liquid chromatography (LC), mass spectrometry (MS)

## Abstract

**Background**: Ovarian cancer (OC) remains one of the most lethal cancers among women due to most cases going undiagnosed until later stages. The early detection and treatment of this malignancy provides the best prognosis, but the lack of an accurate and sensitive screening tool combined with ambiguous symptoms hinders these diagnoses. In contrast, screening for cervical cancer via Papanicolaou (Pap) tests is a widespread practice that greatly reduces the cancer’s mortality rates. Interestingly, previous studies show evidence of OC cells in Pap tests, suggesting that proteins, and potentially lipids, shed from ovarian tumors end up in the cervix. The goal of this study is to evaluate the practicality of using Pap tests as biospecimens for OC-screening-related metabolomics. **Methods**: To evaluate the effectiveness of using residual Pap test samples as biospecimens for potential metabolomics work, 29 Pap test samples, collected from women over the age of 50 with normal cytology and no visible blood contamination, were first obtained from the University of Minnesota, with IRB approval. These samples were centrifuged to recover the cell pellets from the supernatants. The cell pellets underwent a biphasic extraction, followed by an RP-LC-MS analysis, while the supernatants underwent two separate extractions and analyses, including RP-LC-MS and HILIC-LC-MS. Non-targeted features were detected in the range of 220–1000 *m/z* to determine the sensitivity and scope of the various extraction and analytical workflows, as well as evaluating residual Pap test samples as viable metabolomics biospecimens. **Results**: The biphasic extraction and subsequent RP-LC-MS analysis of the isolated cell pellets from all 29 samples yielded informative, exploratory data, highlighting the potential of using residual Pap test samples as biospecimens for metabolomics, specifically lipidomics, studies. Each sample was analyzed in both the positive and negative ion mode, yielding the detection of 7318 in the positive ion mode and 3733 in the negative ion mode. Using multiple reference libraries, 22.85% and 36.19% of these features were annotated in the positive and negative ion mode, respectively. Among these detected features, 453 unique lipids, representative of 20 different lipid subclasses, were annotated in all 29 samples. Of the various lipid subclasses represented from the detected lipids, ceramides, triacylglycerols, hexosylceramides, and phosphatidylcholines contributed to over half (53.3%) of the detected lipids at 16.2%, 13.0%, 12.8%, and 11.3%, respectively. **Conclusions**: The detection of these 453 common lipids across all patients establishes a relative lipidome baseline for women over the age of 50 with normal cervical cytology. This exploratory study is the first investigation to utilize residual Pap test samples as biospecimens in a metabolomics/lipidomics workflow.

## 1. Introduction

The early detection of ovarian cancer (OC) is crucial for improved patient survival and disease outcomes. However, non-invasive, non-surgical tools for the accurate detection of OC in the general population have yet to be developed. In most cases, OC patients experience nonspecific symptoms, including abdominal pain and bloating, and current methods of OC detection lack adequate sensitivity and specificity, leading to disease progression and tumor metastasis [1,2,3]. On the other hand, screening for cervical cancer with Papanicolaou (Pap) tests has been routinely performed on women for over 50 years, reducing the morbidity and mortality associated with it [4,5,6,7].

Currently, there are two types of Pap smear tests: a conventional method in which cells collected from the ectocervix are fixed and stained on a glass slide, and a liquid-based cytology method [8]. In a liquid-based Pap test, cells are collected from the ectocervix and placed in a vial containing an alcohol-based fixative to preserve cells. The fixative is removed from the vials and undergoes automated processing to spread cells evenly on a slide for a pathologist to examine for the presence of premalignant or malignant cells [9]. Strikingly, OC cells have been observed in Pap tests [10,11], indicating that a number of OC biomarkers could also be available for detection in routine Pap tests. In a feasibility study, Boylan et al. previously reported the use of liquid-based Pap test fixatives as a biospecimen amenable for protein identification by mass spectrometry [12]. In later studies, Boylan et al. showed that proteins in the Pap test fixative of a patient with OC were also present in protein extracts from the solid tumor of the same patient [13]. These earlier studies suggest that proteins shed from the ovarian tumor flow through the fallopian tube into the cervical opening. The presence of these proteins provides a rationale to further investigate if any cancer-associated lipids, shed from OC tumors or within the cervix, are also present in these residual Pap test samples. As lipids have previously been reported to play a significant role in cancer progression and tumor metastasis [14,15], the quantitative analysis of the lipidome in these biospecimens is of critical importance. We hypothesize that metabolite/lipid alterations, previously observed in serum and OC tissues, could also be detected in cell pellets extracted from liquid-based Pap tests. As metabolic alterations associated with OC are much more profound in tumor tissues than in serum [16], the use of a biospecimen more proximal to the tumor could improve the probability of detecting OC earlier. To our knowledge, metabolomics or lipidomics studies of Pap test samples for the detection of OC have yet to be reported.

Here, we perform a discovery study to establish the feasibility of using liquid-based Pap tests as biospecimens for metabolomics and lipidomics studies. This cornerstone study is the first step to determine whether it is feasible to use Pap tests as a source for the discovery of OC metabolite or lipid biomarkers. A workflow was developed and optimized to process residual (waste) liquid-based Pap test samples from 29 women with normal cytology. An ultra-high performance liquid chromatography–mass spectrometry (UHPLC-MS) pipeline was developed to analyze the resulting cell pellets, with results suggesting that the rich lipidomics data generated could be a valuable source of biomarkers for cancers affecting the female reproductive system.

## 2. Materials and Methods

### 2.1. Chemicals

LC-MS-grade methanol, 2-isopropanol (IPA), water, and chloroform were purchased from Fisher Chemical (Fisher Scientific, Pittsburgh, PA, USA) and used for sample preparation. LC-MS-grade IPA, water, acetonitrile (ACN), ammonium acetate, ammonium formate, and formic acid (99.5+%) were purchased from Fisher Chemical and used to prepare chromatographic mobile phases. Acid-washed glass beads (500 µm) were purchased from Sigma Aldrich (St. Louis, MO, USA). Isotopically labeled lipid standards were bought from Avanti Polar Lipids (Alabaster, AL, USA) and were used for preparing an in-house lipid internal standard mixture (Table S1).

### 2.2. Clinical Specimens

**Figure 1:**
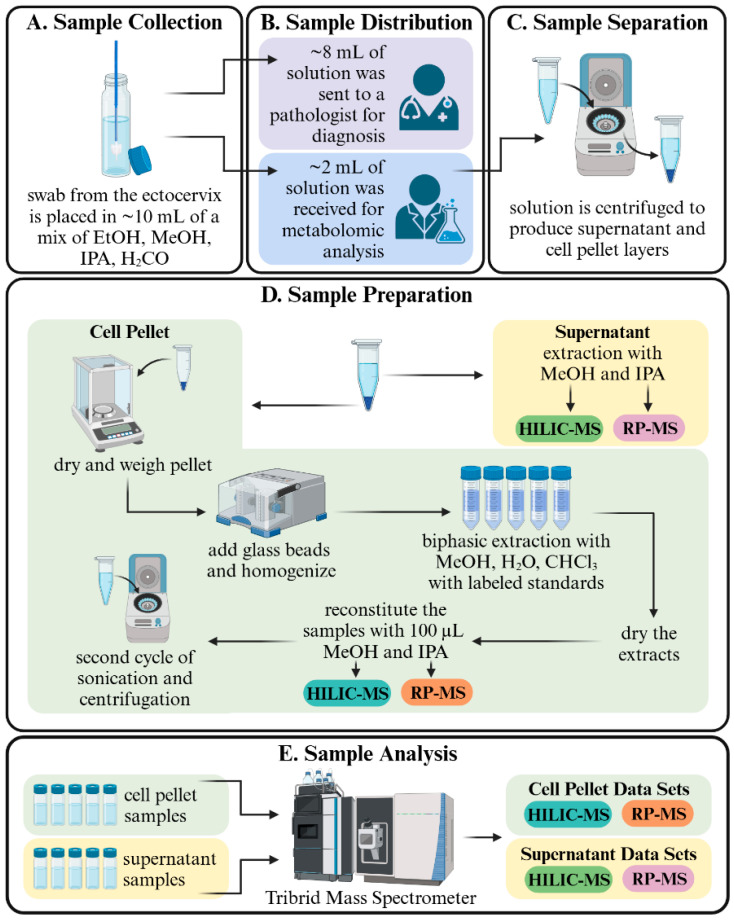
Sample collection and analysis workflow for Pap test metabolomics/lipidomics analysis. (**A**) 29 Pap test samples were collected from women over the age of 50 with no cervical maladies. (**B**) The samples were originally sent to a cytopathologist for diagnosis, and approximately 2 mL of residual samples were received and processed. (**C**) Each sample was separated into a cell pellet and supernatant fraction by centrifugation. (**D**) The cell pellet underwent two different analysis arms: hydrophilic interaction liquid chromatography (HILIC) and reverse-phase (RP) chromatography. (**E**) The extractions from the cell pellet and supernatant samples were placed on the autosampler of Vanquish Horizon UHPLC System coupled to an Orbitrap ID-X Tribrid mass spectrometer for analysis. This figure was created with Biorender.com.

For this study, residual BD Surepath^TM^ solvent from Pap test samples (~2 mL) were obtained from the University of Minnesota BioNet Tissue Procurement Facility with IRB approval (STUDY00016675) and from Dr. Amy Skubitz of the University of Minnesota’s Department of Laboratory Medicine and Pathology. Twenty-nine samples, collected from women (all above 50 years of age) with normal cytology and no visible blood contamination, were used for method development. Samples were stored at 4–5 °C prior to analysis. All samples were de-identified for this study. An overview of the entire sample collection and analysis workflow for this lipidomics study can be found in Figure 1.

### 2.3. Pap Test Sample Collection

Pap samples were collected using BD SurePath^TM^ liquid-based Pap test (Catalog No. 490527, Becton-Dickinson Diagnostics, Franklin Lakes, NJ, USA). Cervical cells were collected from the ectocervix of women using a BD broom-like Cervex brush (Catalog No. 490524-GYN-0500B). Following collection, the detachable head of the Cervex brush was placed into 10 mL of BD SurePath^TM^ solution, comprising 21.7% ethanol, 1.2% methanol, 1.1% isopropanol, and formaldehyde (Figure 1A) [17]. Samples were processed at the University of Minnesota cytopathology clinical laboratory, where 8 mL of the BD SurePath^TM^ solution underwent standard automated processing for diagnosis by a cytopathologist (Figure 1B).

### 2.4. Sample Preparation

The residual BD SurePathTM liquid-based Pap test samples (~2 mL) were transferred to 2 mL microcentrifuge tubes (Figure 1B). Samples were vortexed for 10 s followed by centrifugation at approximately 7130 × *g* (5000 rpm) for 5 min to pellet the cells (Figure 1C). Supernatants were removed and saved for further analysis. Cell pellets were dried in a SpeedVac (Labconco Corporation, Kansas City, MO, USA) and weighed. Six hundred µL of IPA and 0.2 g of 500 µm glass beads were then added to each cell pellet, followed by homogenization in a Tissuelyser (QIAGEN, Germantown, MD, USA). Samples were then re-dried in a SpeedVac, and the metabolites were extracted with a biphasic solution comprising 600 µL of chloroform, 600 µL of methanol, and 300 µL of water. Extracts were sonicated for 5 min and then centrifuged at 21,100 × *g* (14,800 rpm) for 7 min. The chloroform extract from each sample was transferred to a 1.5 mL microcentrifuge tube and dried in the SpeedVac. An extraction mixture was prepared by mixing 50 µL of the isotopically labeled internal standards mixture (Table S2) with 3000 µL of IPA. Eighty µL of this mixture were added to each of the dried chloroform extracts. After IPA extraction, samples underwent a second cycle of sonication for 5 min and centrifugation at 21,100× *g* (14,800 rpm) for 7 min. Cell pellet extracts and supernatant extracts were then transferred to LC-vials for UHPLC-MS analysis (Figure 1D). A blank sample was prepared with LC-MS-grade IPA and underwent the same preparation process as the samples. A pooled quality control (QC) sample was prepared by mixing 3 µL aliquots of each of the sample extracts. Samples were stored at 4–5 °C until analysis.

### 2.5. Ultrahigh Performance Liquid Chromatography–Mass Spectrometry

Reverse-phase (RP) UHPLC-MS was performed with a ThermoFisher Scientific C30, 150 × 2.1 mm, 2.6 µm-particle-size column equipped on a Vanquish Horizon UHPLC system (ThermoFisher Scientific, Waltham, MA, USA). This chromatography system was coupled to an Orbitrap ID-X Tribrid mass spectrometer (ThermoFisher Scientific, Waltham, MA, USA). Hydrophilic interaction liquid chromatography (HILIC) UHPLC-MS was performed with a Waters Corporation Acquity UHPLC BEH Amide 150 × 2.1 mm, 1.7 µm-particle-size column equipped on the same UHPLC-MS system (Figure 1E). The mobile phases used for the RP analysis were as follows. For positive ion mode analyses, mobile phase A was 10 mM ammonium formate in water/acetonitrile (40:60 *v*/*v*) with 0.1% formic acid. Mobile phase B was 10 mM ammonium formate in 2-isopropanol/acetonitrile (90:10 *v*/*v*) with 0.1% formic acid. For negative ion mode analyses, mobile phase A was 10 mM ammonium acetate in water/acetonitrile (40:60 *v*/*v*), and mobile phase B was 10 mM ammonium acetate in 2-isopropanol/acetonitrile (90:10 *v*/*v*). For HILIC positive and negative ion mode analyses, mobile phase A was 10 mM ammonium formate in water/acetonitrile (80:20 *v*/*v*) with 0.1% formic acid, and mobile phase B was acetonitrile with 0.1% formic acid. Further specifics on the LC gradients for both RP and HILIC-UHPLC can be found in the Supporting Information (Tables S3 and S4).

MS data were acquired in both positive and negative ion modes in the 150–2000 *m/z* range. MS/MS experiments were conducted on the pooled QC sample, using the ThermoFisher Scientific AcquireX data acquisition workflow. Precursor ions were fragmented in the high collision dissociation (HCD) cell with stepped collision energy of 15%, 50%, and 50% and were sequentially fragmented with a collision-induced dissociation of 40%.

### 2.6. Data Analysis

**Figure 2:**
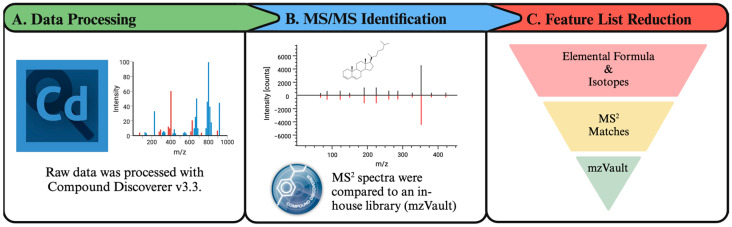
Data processing workflow for Pap test metabolomics/lipidomics analysis. (**A**) The raw data files were processed with ThermoFisher Scientific Compound Discoverer v3.3. (**B**) The same software was used for MS/MS annotation with ThermoFisher Scientific’s mzCloud and in-house mzVault library. (**C**) These steps increased the annotation confidence while reducing false positives. This figure was created with Biorender.com.

Raw data were processed with ThermoFisher Scientific Compound Discoverer v3.3. This process involved blank feature filtering, retention time alignment, peak picking and peak area integration, and isotopic feature grouping, as well as adduct grouping, and compound area correction using the QC based regression curve. An overview of the LC-MS data processing workflow that was followed is given in Figure 2.

The annotated lipids from both positive and negative ion modes were first examined for all 29 samples in our patient cohort to identify which annotated lipids were common to every sample. These common, annotated lipids were then normalized by lipid subclass according to the total number of annotated lipids detected from the entire patient cohort. A separate, second normalization step was taken to normalize the data by lipid subclass per patient sample. This included normalizing the lipid subclass of a single sample according to the total number of annotated lipids detected from that specific sample. No outliers were removed from this dataset.

## 3. Results

### 3.1. Reverse-Phase UHPLC-MS Analysis of Cell Pellets from Residual Pap Test Fluid Enables Significant Lipidome Coverage

To determine the approach that would provide the largest depth of coverage for the normal Pap test sample set, all 29 patient samples’ supernatants and cell pellets were examined individually. Each supernatant was divided into two aliquots that underwent two different treatments: an IPA extraction followed by reverse-phase liquid chromatography (RP-LC) and an MeOH extraction followed by liquid chromatography equipped with an HILIC column. The individual, isolated cell pellets were weighed, with an average weight of 1.0871 g (*n* = 29, 1.0835–1.0909). Each pellet then underwent a biphasic extraction followed by RP UHPLC-MS as described above. Both cell pellets and supernatants were analyzed via both RP UHPLC-MS and HILIC UHPLC-MS. LC-MS data were processed in ThermoFisher Scientific Compound Discoverer v3.3 to extract aligned “compounds” common to the various samples. The number of compounds detected for either the supernatants or the cell pellets is given in Table 1.

The biphasic extraction and subsequent analysis of the cell pellet via RP UHPLC-MS achieved the largest depth of coverage as seen by the number of compounds resulting from the Compound Discoverer data analysis (Table 1). LC-MS positive and negative ion mode cell pellet datasets yielded 7318 and 3733 de-isotoped, de-adducted compounds, respectively (Table 2). The number of detected compounds in the two supernatant aliquots that underwent MeOH or IPA extractions was marginal in comparison to those detected in the cell pellets (Table 1). The compounds annotated in the cell pellet dataset were mostly lipids, as expected by the polarity of the extraction solvents used. A further in-depth analysis of the cell pellet dataset was pursued. Supernatant data were not further investigated.

The differences observed in the number of detected features between each ion mode were expected, as more species are often detected in the positive ion mode due to its greater sensitivity and lower specificity. As stated previously, annotations were performed in Compound Discoverer v3.3 with reference to ThermoFisher Scientific’s mzCloud library and an in-house mzVault library developed for the Molecular Transducers of Physical Activity Consortium [18]. Tandem MS data collection was performed via ThermoFisher Scientific’s AcquireX workflow. While some annotations for small metabolites were made, only the annotated lipids (233 in the positive ion mode and 265 in the negative ion mode) were preserved for further analysis to try and establish a baseline normal Pap cell lipidome from our human subject cohort. It is highly likely that many additional lipids were present among the unannotated compounds, but the annotation of those was outside the scope of this first study.

### 3.2. Lipidome Composition of Normal Pap Cell Pellets

**Figure 3:**
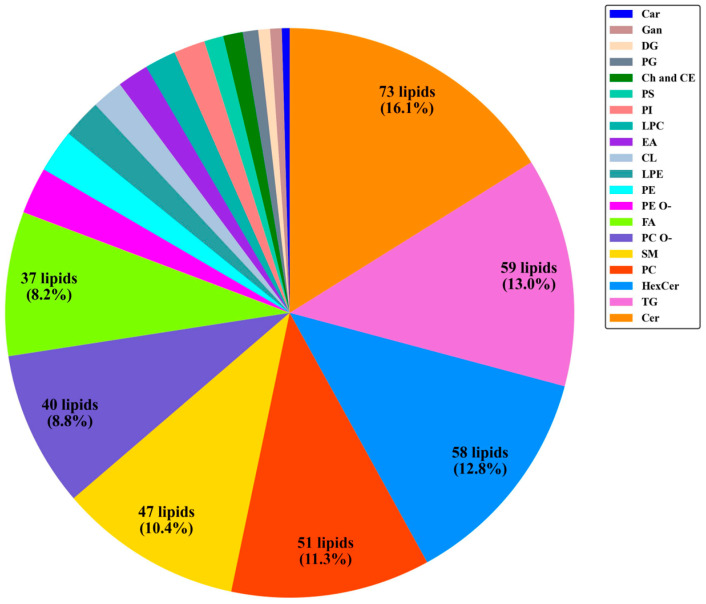
Distribution of lipids annotated in Pap cell pellet samples. A total of 453 unique lipid species were annotated across 20 lipid subclasses. Lipid subclass HexCer includes HexCer (10.37%), Hex2Cer (1.54%), and Hex3Cer (0.88%). The lipid subclass name corresponding to each abbreviation can be found in Table S1.

To obtain an overview of the average Pap cell lipidome of women over the age of 50 with normal cervical cytology, we applied our RP UHPLC-MS method to the 29 cell pellets. Each cell pellet’s lipidome was evaluated individually, and each lipid annotation was confirmed via MS/MS experiments conducted on pooled QC samples. A total of 453 unique lipids were annotated across all samples in positive and negative ion modes. These annotated lipids were assigned to 20 different lipid subclasses (Table 3 and Figure 3). Information regarding each individual annotated lipid species is found in Table S5. Of the 20 lipid subclasses that were detected, the lipids Cer, TG, HexCer, and PC contributed to over half (53.3%) of the annotated lipids at 16.2%, 13.0%, 12.8%, and 11.3%, respectively (Figure 3). Furthermore, the lipid subclasses of SM, PC O-, and FA contributed to an additional ~28% to the total number of annotated lipids. The remaining 13 lipid subclasses constituted only ~20% of the lipids that were annotated as listed in Table 3.

**Figure 4:**
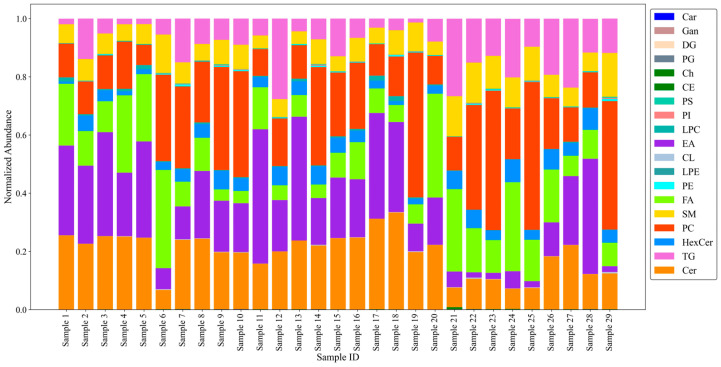
Lipidomics profile of individual Pap test cell pellets. Normalized total lipid abundances for the 20 detected lipid subclasses. Total abundance for each lipid class was calculated by summing the relative abundances of all annotated lipids in that class. For each sample, the relative abundance of the lipid species was normalized to the total lipid abundance per sample. For visualization purposes, lipid subclasses PE O-, PC O-, LPE O-, and LPC O- are included under subgroups PE, PC, LPE, and LPC, respectively. Lipid subclass HexCer includes HexCer, Hex2Cer, and Hex3Cer. The lipid subclass name corresponding to each abbreviation can be found in Table S1.

The cell pellet of each patient was also examined individually; the distributions of various lipid classes unique to each sample are given in Figure 4. Overall, the abundances of many of the lipid classes were rather similar. For example, Cer, EA, and PC were present in relatively high abundance in many of the patient’s Pap test cell pellets (Figure 4); SM and HexCer values were relatively similar among the samples. Several of the other lipids varied significantly between patients (e.g., TG and FA), a finding that determines the baseline above which any candidate biomarkers will have to be identified. This lipidome variability was somewhat expected due to the complexities in the makeup of an individual’s metabolome/lipidome. A further direct comparison of the patient’s lipidomes was not possible due to the lack of metadata as per the IRB’s de-identification regulations.

**Figure 5:**
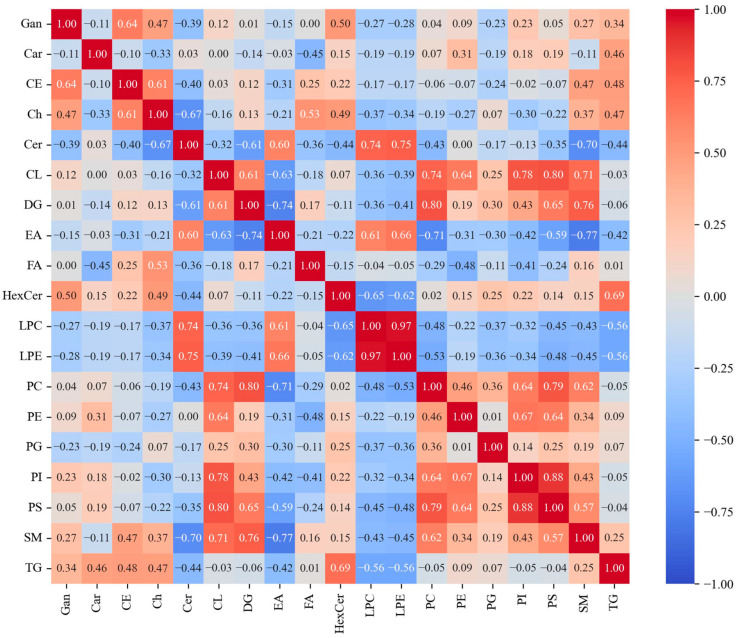
Correlation map of lipids annotated in Pap test samples. Positively and negatively correlated normalized total lipid abundances for the 20 detected lipid subclasses. For visualization purposes, lipid subclasses PE O-, PC O-, LPE O-, and LPC O- are included under subgroups PE, PC, LPE, and LPC, respectively. Lipid subclass HexCer includes HexCer, Hex2Cer, and Hex3Cer. The lipid subclass name corresponding to each abbreviation can be found in Table S1.

The positive and negative correlations of individual lipid subclasses were examined using the normalized lipid abundances (Figure 5). While the vast majority of the correlations were neither positive nor negative, some significant correlations were noted, mainly amongst many of the lipids associated with glycerophospholipid metabolism. Strong, positive correlations were observed between most of the phospholipids, particularly LPC, LPE, PC, PI, and PS. Other moderately strong, positive correlations were observed between the various phospholipids and the CL and DG lipid subclasses. The only significantly negative correlations observed were between the EA and SM subclasses and between the EA and DG subclasses. The correlation between the SM and Cer subclasses was also moderately negative.

## 4. Discussion

This study represents the first attempt to describe the metabolome, specifically the lipidome, of residual BD SurePath^TM^ Pap fluid samples from female patients with normal cervical cytology. Across the 29 samples in our cohort, 453 unique lipids were annotated in total across every sample, establishing a relative lipid profile baseline for women over the age of 50 with no cervical maladies. This preliminary study complements and builds on the work previously carried out by Boylan et al., which found that residual Pap test fluid samples contain enough proteins for a proteomics analysis [13]. The presence of these proteins, in addition to our discovery of numerous lipids, highlights the potential use of residual Pap test samples as a valuable biospecimen to identify biomarkers indicative of various gynecological maladies.

Following the exploration of the depth of coverage produced by various LC-MS methods in the different fraction obtained from residual Pap samples, the focus of this study centered on the abundant cell pellet derived from the BD SurePath^TM^ solution due to the significantly higher number of metabolites detected in both positive and negative ion modes as seen in Table 1. An RP UHPLC-MS analysis of these cell pellets allowed for the detection of some of the most abundant lipid subclasses, including ceramides, triacylglycerols, phosphatidylcholines, sphingomyelins, and fatty acids, as seen in Figure 3. The lipid profile determined from the Pap test samples coincided with previous literature results regarding the lipid makeup of non-disease cervical and epithelial cells. Preetha et al. have reported that sphingomyelins are the most abundant lipid subclass present in non-diseased cervical tissue samples [19], and, since ceramides are a direct product of the dephosphorylation process of sphingomyelins, our findings of abundant ceramides support this conclusion. The same study also quantified the concentrations of five phospholipid subclasses, including phosphatidylcholines, phosphatidylethanolamines, phosphatidylinositols, phosphatidylglycerols, and phosphatidylserines, in healthy cervical tissue samples [19]. The measured concentrations of these lipids are in agreement with the number of detected lipids of the same phospholipid subclasses found in our analysis (Figure 3). These same six subclasses of phospholipids, in addition to sphingomyelins, showed significantly positive correlations with one another and with the cardiolipin and diacylglycerol subclasses (Figure 5), suggesting shared functional relationships and regulatory pathways between these specific subclasses. These eight phospholipid subclasses are well-known to be intrinsically linked by the glycerophospholipid and sphingolipid metabolic pathways [20], so these positive correlations are expected.

Another characteristic of non-diseased cervical tissue is the presence of lipid droplets (LDs), which are cellular organelles that are a source of energy storage and lipid metabolism within cells [21,22]. As LDs are predominantly made up of triacylglycerols and steryl esters surrounded by a phospholipid monolayer, the observation of abundant triacylglycerols and other phospholipids, such as phosphatidylcholines and sphingomyelins, within the cell pellets is also in agreement with previous literature findings [15,22]. The concurrent detection of fatty acids, the foundational building blocks of most lipid species, is also expected as these lipids play critical roles in membrane synthesis, energy storage, and cell signaling, as well as other cellular processes [16]. As illustrated in Figure 4, the distributions of the many lipid subclasses varied between individual samples, which demonstrates the biological variability between samples that can originate from differences in each patient’s diet, lifestyle, genetics, and other environmental causes. With this variability in mind, the presence of these specific lipid subclasses across all 29 Pap test cell pellets is likely indicative of healthy epithelial cells and serves as a relative baseline for the non-diseased cervical lipidome of female patients over the age of 50.

The dysregulation of lipid synthesis and the associated lipidome abundance abnormalities are now widely accepted as a core signature of cancer cell proliferation [23]. These lipid alterations are associated with the activation of several oncogenic pathways, leading to significant lipid accumulation, contributing to carcinogenesis and tumor metastasis [23]. Significant differences between lipid profiles among patients with cervical cancer and healthy controls have been found in both cervical scrapings and plasma samples [19,24,25]. Preetha et al. found the concentrations of all six investigated phospholipid subclasses, including phosphatidylcholines, phosphatidylethanolamines, phosphatidylinositols, phosphatidylglycerols, sphingomyelins, and phosphatidylserines, were significantly increased when comparing cancerous samples versus healthy controls [19]. Protein OC biomarkers have also been found to be present in BD SurePath^TM^ residual Pap test fluid [12], highlighting the possibility of proteins and other small molecules being shed by ovarian tumors through the fallopian tubes into the uterus. Another emerging hallmark of cancer development, including breast, lung, and colorectal cancers, is mitochondrial dysfunction related to perturbations in the concentrations of deuterium in metabolic matrix water [26,27,28]. Previous work carried out by Arima et al. identified dysregulated metabolic pathways via the reduction in levels of alpha-ketoglutarate as well as the elevated concentrations of branched amino acids, including leucine and isoleucine, all in colorectal tumor tissues [27]. The elevations of these amino acids and their degraded metabolites of acetyl-CoA and succinyl-CoA point to mitochondrial dysfunction and hindered the transfer of protons to metabolic matrix water [29]. This lack of deuterium transfer to the metabolic matrix water leads to the continuous accumulation of deuterium within cells, which has been found to lead to cancer development [29]. These previous findings of elevated levels of various phospholipid subclasses in diseased cervical tissue compared to healthy cervical tissue and observations of depleted levels of alpha-ketoglutarate and elevated levels of leucine and isoleucine, respectively, in other types of cancer warrant further targeted and non-targeted investigation in our future work that will include both cancerous Pap test samples and non-diseased controls.

In these same future studies, we also plan to address the potential bias in the extracted lipid composition arising from our choices of extraction solvents. As Reis et al. have found when comparing multiple extraction solvents, including the Folch, Bligh and Dyer, methanol-tert-butyl methyl ether, and hexane-isopropanol methods, the detected levels of certain lipid subclasses varied depending on which extraction protocol was used [30]. The concentrations of the predominantly detected lipid subclasses, like cholesterol esters, triacylglycerols, and phosphatidylcholines, varied only slightly between extraction methods while the concentrations of less prevalent lipid subclasses, such as ceramides, phosphatidylinositols, and lyso-phosphatidylcholines, were found to be increased following only the Folch and acidified Bligh and Dyer protocols [30]. Due to the presence of these variations that are dependent on the respective extraction method, we plan to investigate and compare the potential differences of these methods in the lipid compositions of our samples in future studies.

Our findings provide a rationale to further investigate the lipidome differences between OC Pap test samples and those from healthy patients or patients with benign conditions in future studies. However, it is still important to point out the limitations of our current findings. Our dataset across more than two dozen female subjects clearly showed normal population heterogeneity, and it is critical to capture this diversity prior to establishing any comparisons to cancerous Pap test cell pellet samples. We are aware, however, that it will be necessary to analyze a substantial number of healthy subject samples selected from a more diverse population of women. The patient cohort studied here comprised only a small subset of patients with normal cervical cytology over 50 years of age, from the Midwest region of the United States. A more representative sample pool, spanning various ethnicity subgroups, ages, and disease stages, as well as age-matched healthy control samples of a similar, diverse nature will likely be needed in any biomarker discovery studies involving Pap test cell pellets. A full understanding of the normal population heterogeneity will be critical in establishing statistically significant comparisons against cancer patients. Comparisons between cervical and ovarian cancer conditions should likely also be undertaken to determine the specificity of any lipidomics profiles found in Pap test cell pellets. To make our future experimental processes more applicable for clinical settings, we will investigate using a microscopy and mass spectrometry imaging (MSI) avenue to establish a more realistic diagnostic workflow that would include a preliminary screening via microscopy, followed by more extensive investigation into the differences in cell structure via MSI and in small molecule composition via RP-UHPLC-MS.

## 5. Conclusions

The annotation of 453 unique lipids, representative of 20 subclasses of lipids, present in all 29 samples within our patient cohort confidently establishes a preliminary baseline Pap test cellular lipid profile for women over the age of 50 with no cervical maladies. The vast majority of the useful information, from a lipidomics perspective, was found in the cell pellet component of residual BD SurePath^TM^ Pap fluid, which yielded rich chromatograms when a careful sample preparation workflow was developed. Since millions of Pap tests are administered every year during annual gynecology exams, a lipidomics analysis of Pap test cell pellets will likely become a valuable source of metabolic information that could potentially aid in early and non-invasive diagnoses of OC and other gynecological malignancies.

## Figures and Tables

**Table 1:** Features detected from the analysis of different Pap sample fractions. Number of compounds collected via reverse-phase liquid chromatography or hydrophilic interaction liquid chromatography, in both positive and negative ion modes.

Studied Fraction	Positive Ion Mode	Negative Ion Mode
HILIC Supernatant	58	75
RP Supernatant	56	46
HILIC Cell Pellet	256	2
RP Cell Pellet	7318	3733

**Table 2:** Breakdown of the compounds resulting from Compound Discoverer analysis of the samples’ cell pellet LC-MS data. Number of compounds, collected via reverse-phase liquid chromatography, in both positive and negative ion modes, including percentages of compounds annotated and compounds with MS/MS data.

Compound Subsets	Positive Ion Mode	Negative Ion Mode
Annotated Compounds	1672	1351
Unannotated Compounds	5646	2382
Compounds with MS/MS Data	3447	2382
Total Compounds	7318	3733
% Annotated	22.85%	36.19%
% with MS/MS Data	47.10%	63.71%

**Table 3:** Number of lipids detected per lipid subclass from highest to lowest abundance. Number of lipids per lipid subclass and percentage of each subclass’ contribution to the total lipid count in descending order as illustrated in Figure 3. Lipid subclass abbreviation and full name are also included.

Lipid Abbreviation	Lipid Name	Number of Lipids	Percentage of Total
Cer	Ceramides	73	16.1
TG	Triacylglycerols	59	13.0
HexCer	Hexosylceramides	58	12.8
PC	Phosphatidylcholines	51	11.3
SM	Sphingomyelins	47	10.4
PC O-	Ether Phosphatidylcholines	40	8.8
FA	Fatty Acids	37	8.2
PE O-	Ether Phosphatidylethanolamines	12	2.6
PE	Phosphatidylethanolamines	11	2.4
LPE	Lysophosphatidylethanolamines	9	2.2
CL	Cardiolipins	8	1.8
EA	Fatty Amides	8	1.8
LPC	Lysophosphatidylcholines	8	1.8
PI	Phosphatidylinositols	5	1.1
PS	Phosphatidylserines	5	1.1
Ch and CE	Cholesterol and Cholesterol Esters	5	1.1
PG	Phosphatidylglycerols	4	0.9
DG	Diacylglycerols	3	0.7
Gan	Gangliosides	3	0.7
Car	Carnitines	2	0.4

## Data Availability

The data obtained in this study will be accessible at the National Institute of Health’s Common Fund’s NMDR (supported by the NIH grant, U01-DK097430) website, the Metabolomics Workbench, https://www.metabolomicsworkbench.org/, with the study ID ST005268.

## Supplementary Materials

The following supporting information can be downloaded at: https://www.mdpi.com/article/10.3390/metabo14110600/s1, Table S1: Lipid subclass abbreviations; Table S2: Isotopically labeled lipid standards; Table S3: Liquid chromatography gradient for reverse-phase LC-MS; Table S4: Liquid chromatography gradient for hydrophilic interaction liquid chromatography LC-MS; Table S5: Details for the 453 annotated lipids in Pap test cell pellets.
