# Supplementary material for: Metabolomics of Papanicolaou Tests for the Discovery of Ovarian Cancer Biomarkers
Source: Metabolites. 2024 Nov 7;14(11):600. doi: 10.3390/metabo14110600 (PMC11596055; doi:10.3390/metabo14110600)
Supplement: Supplementary file 1 [file metabolites-14-00600-s001.zip › metabolites-3281333-supplementary.pdf]

Table S1. Lipid Subclass Abbreviations. Lipid subclass names in correspondence to their common letter abbreviation.

Lipid Subclass Abbreviation	Lipid Subclass Name
CE	Cholesterol Esters
CL	Cardiolipins
DG	Diacylglycerols
LPC	Lysophosphatidylcholines
LPE	Lysophosphatidylethanolamines
PC	Phosphatidylcholines
PE	Phosphatidylethanolamines
PG	Phosphatidylglycerols
PI	Phosphatidylinositols
PS	Phosphatidylserines
SM	Sphingomyelins
TG	Triacylglyercols

Table S2. Isotopically Labeled Lipid Standards. Lipid names and CAS numbers for the isotopically labeled standards purchased from Avanti Polar Lipids are reported. The internal standard stock solution was made in-house, and the respective concentrations of each lipid are reported. These standards aided in quantification and standardization of our reverse-phase UHPLC-MS method.

Lipid Subclass Name	Isotopically Labeled Lipids	CAS Number	Concentration (μg mL^{-1})
Cholesterol Ester	CE (18:1(d7))	1416275-35-7	350
Cholesterol	Cholesterol-d7	83199-47-7	100
Diacylglycerol	DG (15:0/18:1(d7))	2097561-14-1	10
Lysophosphatidylcholine	LPC (18:1(d7))	2097561-13-0	25
Lysophosphatidylethanolamine	LPE (18:1(d7))	2260669-47-2	5
Phosphatidylcholine	PC (15:0/18:1(d7))	2097561-16-3	160
Phosphatidylethanolamine	PE (15:0/18:1(d7))	2097561-15-2	5
Phosphatidylglycerol	PG (15:0/18:1(d7))	2260669-42-7	30
Phosphatidylinositol	PI (15:0/18:1(d7))	2260669-44-9	20
Phosphatidylserine	PS (15:0/18:1(d7))	2260669-40-5	10
Sphingomyelin	SM (d18:1/(d9))	2260669-50-7	30
Triacylglycerol	TG (15:0/18:1(d7)/15:0)	2097561-17-4	55

Table S3. Liquid Chromatography Gradient for Reverse-Phase LC-MS. For positive ion mode analysis, mobile phase A was 10 mM ammonium formate in water/acetonitrile (40:60 v/v) with 0.1% formic acid. Mobile phase B was 10 mM ammonium formate with 2-isopropanol/acetonitrile (90:10 v/v) and 0.1% formic acid. For negative ion mode analyses, mobile phase A was 10 mM ammonium acetate in water/acetonitrile (40:60 v/v), and mobile phase B was 10 mM ammonium acetate in 2-isoproponal/acetonitrile (90:10 v/v). The flow rate on the column was held constant at 0.400 mL/min for the duration of the run. Column temperature was 50°C.

No.	Time	%B	Curve
**1**	0.000	Equilibration	
**2**	0.000	20.0	5
**3**	*New Row*		
**4**	0.000	Run	
**5**	0.000	20.0	5
**6**	1.000	60.0	6
**7**	5.000	70.0	6
**8**	5.500	85.0	6
**9**	8.000	90.0	6
**10**	8.200	100.0	6
**11**	10.500	100.0	6
**12**	10.700	20.0	6
**13**	12.000	20.0	6
**14**	*New Row*		
**15**	12.000	Stop Run	

Table S4. Liquid Chromatography Gradient for Hydrophilic Interaction Chromatography Separation Method. For positive and negative ion modes, mobile phase A was 10mM ammonium formate in water/acetonitrile (80:20 v/v) with 0.1% formic acid, and mobile phase B was acetonitrile with 0.1% formic acid. The flow rate on the column was held constant at 0.400 mL/min for the duration of the run. Column temperature was 40°C.

No.	Time	%B	Curve
**1**	0.000	Equilibration	
**2**	0.000	95.0	5
**3**	*New Row*		
**4**	0.000	Run	
**5**	0.000	95.0	5
**6**	0.500	95.0	6
**7**	8.000	40.0	6
**8**	9.400	40.0	6
**9**	9.500	95.0	6
**10**	12.000	95.0	6
**11**	*New Row*		
**12**	12.000	Stop Run	

Table S5. Details for the 453 annotated lipids in Pap test cell pellets. Lipid number, class, proposed annotation, adduct, experimental monoisotopic *m/z* value, retention time, and annotation confidence level are provided for all 453 detected lipids with possible detection of some in-source fragments. Annotation confidence level was assigned based on the following criteria: 1) MS1 and MS/MS spectrum of standard matched to the compound. 2) MS1 and MS/MS spectrum of the feature matched with library spectra 3) putative ID assignment based on elemental formula match with literature. 4) unknowns.

#	Class	Annotation	Adduct	*m/z*	RT [min]	Annotation Confidence Level
1	Car	Car(14:0)	$[M+H]^+$	372.3108	1.6	3
2	Car	Car(18:0)	$[M+H]^+$	428.3734	2.4	2
3	CE	CE(18:1)	$[M+NH_4]^+$	668.635	9.7	2
4	CE	CE(20:4)	$[M+NH_4]^+$	690.6188	9.3	2
5	Cer	Cer(d17:1/16:0)	$[M+H]^+$	506.4934	4.7	2
6	Cer	Cer(d18:0/16:0)	$[M+H]^+$	540.5355	5.5	2
7	Cer	Cer(d18:0/18:1)	$[M+H]^+$	566.5511	5.0	2
8	Cer	Cer(d18:0/20:0)	$[M+H]^+$	596.5976	6.8	2
9	Cer	Cer(d18:0/22:0)	$[M+H]^+$	624.6288	7.2	2
10	Cer	Cer(d18:0/22:1)	$[M+H]^+$	622.6132	6.8	2
11	Cer	Cer(d18:0/24:0)	$[M+H]^+$	652.6607	7.7	2
12	Cer	Cer(d18:0/24:0)	$[M+H]^+$	652.6606	7.5	2
13	Cer	Cer(d18:0/26:0)	$[M+H]^+$	680.6918	7.9	2
14	Cer	Cer(d18:0/26:0)	$[M+H]^+$	680.6919	8.0	2
15	Cer	Cer(d18:0/28:1)	$[M+H]^+$	706.7079	7.8	2
16	Cer	Cer(d18:0/30:1)	$[M+H]^+$	734.7391	8.6	2
17	Cer	Cer(d18:1/16:0)	$[M+H]^+$	520.5091	5.2	2
18	Cer	Cer(d18:1/16:1)	$[M+H]^+$	520.5092	4.2	2
19	Cer	Cer(d18:1/21:0)	$[M+H]^+$	608.5982	6.6	2
20	Cer	Cer(d18:1/22:0-OH)	$[M+H]^+$	638.6085	4.7	2
21	Cer	Cer(d18:1/23:0)	$[M+H]^+$	636.629	7.2	2
22	Cer	Cer(d18:1/24:0)	$[M+H]^+$	650.645	7.4	2
23	Cer	Cer(d18:1/24:0-OH)	$[M+H]^+$	666.6403	7.2	2
24	Cer	Cer(d18:1/24:1)	$[M+H]^+$	648.6295	7.0	2
25	Cer	Cer(d18:1/25:0)	$[M+H]^+$	664.661	7.6	2
26	Cer	Cer(d18:1/25:1)	$[M+H]^+$	662.6454	7.1	2
27	Cer	Cer(d18:1/26:0)	$[M+H]^+$	678.6762	7.8	2
28	Cer	Cer(d18:1/26:0)	$[M+H]^+$	678.6762	7.5	2
29	Cer	Cer(d18:1/26:1-OH)	$[M+H]^+$	692.6557	7.0	2

30	Cer	Cer(d18:2/24:0)	$[M+H]^+$	648.6291	6.8	2
31	Cer	Cer(d32:0)	$[M+H]^+$	512.504	4.0	2
32	Cer	Cer(d32:1)	$[M-H]^-$	544.4505	4.7	2
33	Cer	Cer(d33:0)	$[M+CH_3COOH-H]^-$	584.5264	5.4	2
34	Cer	Cer(d34:0)	$[M-H]^-$	538.5208	6.1	2
35	Cer	Cer(d35:0)	$[M+CH_3COOH-H]^-$	612.5576	6.3	2
36	Cer	Cer(d35:1)	$[M+CH_3COOH-H]^-$	610.5419	6.1	2
37	Cer	Cer(d36:0)	$[M+CH_3COOH-H]^-$	626.5733	6.6	2
38	Cer	Cer(d36:1)	$[M+CH_3COOH-H]^-$	624.5576	6.5	2
39	Cer	Cer(d37:0)	$[M+CH_3COOH-H]^-$	640.5889	6.8	2
40	Cer	Cer(d37:1)	$[M+CH_3COOH-H]^-$	638.5734	6.7	2
41	Cer	Cer(d38:1)	$[M+CH_3COOH-H]^-$	652.589	6.8	2
42	Cer	Cer(d38:1-OH)_or_Cer(t38:1)	$[M+H]^+$	610.5772	5.8	2
43	Cer	Cer(d38:2)	$[M+CH_3COOH-H]^-$	590.5522	6.6	2
44	Cer	Cer(d39:0)	$[M+CH_3COOH-H]^-$	668.6207	7.1	2
45	Cer	Cer(d40:0)	$[M+CH_3COOH-H]^-$	682.6361	7.1	2
46	Cer	Cer(d40:0-OH)_or_Cer(t40:0)	$[M+H]^+$	640.6241	5.6	2
47	Cer	Cer(d40:2)	$[M+CH_3COOH-H]^-$	696.6153	6.7	2
48	Cer	Cer(d40:2)	$[M+CH_3COOH-H]^-$	678.6047	6.9	2
49	Cer	Cer(d41:0)	$[M+CH_3COOH-H]^-$	696.6517	7.4	2
50	Cer	Cer(d42:0)	$[M+CH_3COOH-H]^-$	710.6676	7.6	2
51	Cer	Cer(d42:0-OH)_or_Cer(t42:0)	$[M+H]^+$	668.6556	7.2	2
52	Cer	Cer(d42:1)	$[M+CH_3COOH-H]^-$	648.6301	7.2	2
53	Cer	Cer(d42:2)	$[M+CH_3COOH-H]^-$	706.6361	7.1	2
54	Cer	Cer(d42:3)	$[M+H]^+$	646.6138	6.8	2

55	Cer	Cer(d43:0)	$[M+CH_3COOH-H]^-$	664.6617	7.7	2
56	Cer	Cer(d43:1)	$[M+CH_3COOH-H]^-$	722.6673	7.6	2
57	Cer	Cer(d44:0)	$[M+CH_3COOH-H]^-$	738.6988	7.9	2
58	Cer	Cer(d44:1)	$[M+CH_3COOH-H]^-$	736.6834	7.8	2
59	Cer	Cer(d44:1-OH)_or_Cer(t44:1)	$[M+CH_3COOH-H]^-$	692.6568	7.3	2
60	Cer	Cer(d44:1-OH)_or_Cer(t44:1)	$[M+CH_3COOH-H]^-$	692.6567	7.6	2
61	Cer	Cer(d44:2)	$[M-H]^-$	734.6674	7.4	2
62	Cer	Cer(d44:2)	$[M-H]^-$	674.646	7.5	2
63	Cer	Cer(d44:3)	$[M+CH_3COOH-H]^-$	732.6518	7.1	2
64	Cer	Cer(d45:1)	$[M+CH_3COOH-H]^-$	750.6985	8.0	2
65	Cer	Cer(d45:2)	$[M+CH_3COOH-H]^-$	748.6829	7.6	2
66	Cer	Cer(t18:0/22:0)	$[M+H]^+$	640.6243	7.0	2
67	Cer	Cer(t18:0/24:0)	$[M+H]^+$	668.6555	7.0	2
68	Cer	Cer(t18:0/24:1)	$[M+H]^+$	666.6398	6.6	2
69	Cer	Cer(t18:0/24:1)	$[M+H]^+$	666.64	5.6	2
70	Cer	Cer(t18:0/25:1)	$[M+H]^+$	680.6557	7.4	2
71	Cer	Cer(t39:0)	$[M+CH_3COOH-H]^-$	684.6153	7.0	2
72	Cer	Cer(t41:1)	$[M+H]^+$	652.6242	6.2	2
73	Cer	Cer(t41:1)	$[M+CH_3COOH-H]^-$	710.6309	6.9	2
74	Cer	Cer(t42:1)	$[M-H]^-$	664.6255	7.3	2
75	Cer	Cer(t42:1)	$[M+CH_3COOH-H]^-$	706.6359	7.0	2
76	Cer	Cer(t42:2)	$[M+CH_3COOH-H]^-$	722.6311	6.7	2
77	Cer	Cer(t43:1)	$[M+CH_3COOH-H]^-$	738.6623	7.1	2
78	Ch	cholesterol	$[M+H-H_2O]^+$	369.3516	4.9	2
79	CE	cholesterol ester	$[M+H-H_2O]^+$	369.3516	9.5	2
80	CE	cholesterol ester	$[M+H-H_2O]^+$	369.3516	9.4	2

81	CL	CL(18:1/18:2/18:2/18:2)	$[M-H]^-$	1449.981	7.3	2
82	CL	CL(18:2/18:2/18:2/18:2)	$[M-H]^-$	1447.965	7.2	2
83	CL	CL(68:6)	$[M-H]^-$	1395.933	7.1	3
84	CL	CL(70:4)	$[M-H]^-$	1427.999	3.9	3
85	CL	CL(70:5)	$[M-H]^-$	1425.98	7.5	3
86	CL	CL(70:7)	$[M-H]^-$	1421.95	7.2	2
87	CL	CL(72:6)	$[M-H]^-$	1451.997	7.5	3
88	CL	CL(74:8)	$[M-H]^-$	1475.996	7.4	3
89	CL	CL(74:9)	$[M-H]^-$	1473.981	7.3	3
90	DG	DG(38:4)	$[M+NH_4]^+$	662.5723	6.6	2
91	DG	DG(18:0/20:4)	$[M+H-H_2O]^+$	627.5352	4.5	2
92	DG	DG(38:3)	$[M+CH_3COOH-H]^-$	705.568	6.9	2
93	EA	EA(17:0)	$[M+H]^+$	314.3054	2.5	2
94	EA	EA(17:0)	$[M+H]^+$	314.3054	1.4	2
95	EA	EA(18:0)	$[M+H]^+$	328.3211	2.1	2
96	EA	EA(20:0)	$[M+H]^+$	356.3522	2.0	2
97	EA	EA(20:0)	$[M+H]^+$	356.3523	2.6	2
98	EA	EA(20:3)	$[M+H]^+$	350.3054	1.3	2
99	EA	EA(22:0)	$[M+H]^+$	384.3834	2.3	2
100	EA	EA(22:0)	$[M+H]^+$	384.3835	2.3	2
101	FA	FA(16:0)	$[M-H]^-$	255.2329	2.5	2
102	FA	FA(16:0-OH)	$[M-H]^-$	271.2278	1.3	2
103	FA	FA(16:0-OH)	$[M-H]^-$	271.2279	1.8	2
104	FA	FA(16:1)	$[M-H]^-$	253.2173	2.2	2
105	FA	FA(17:0)	$[M-H]^-$	269.2486	2.7	2
106	FA	FA(17:1)	$[M-H]^-$	267.233	2.4	2
107	FA	FA(18:0)	$[M-H]^-$	283.2642	3.0	2
108	FA	FA(18:0-OH)	$[M-H]^-$	299.2592	1.9	2
109	FA	FA(18:0-OH)	$[M-H]^-$	299.2592	1.8	3
110	FA	FA(18:1)	$[M-H]^-$	281.2486	2.6	2
111	FA	FA(18:2)	$[M-H]^-$	279.233	2.3	2
112	FA	FA(18:2-2OH)	$[M-H]^-$	311.2228	1.2	2
113	FA	FA(19:0)	$[M-H]^-$	297.2799	3.3	2
114	FA	FA(20:1)	$[M-H]^-$	309.2799	3.0	2
115	FA	FA(20:2)	$[M-H]^-$	307.2643	2.6	2
116	FA	FA(20:3)	$[M-H]^-$	305.2487	2.4	2

117	FA	FA(20:3)	[M-H]$^-$	305.2486	2.5	2
118	FA	FA(20:4)	[M-H]$^-$	303.233	2.3	2
119	FA	FA(20:5)	[M-H]$^-$	301.2173	2.1	2
120	FA	FA(21:0)	[M-H]$^-$	325.3113	4.1	2
121	FA	FA(21:0)	[M-H]$^-$	325.3113	3.8	2
122	FA	FA(22:0-OH)	[M-H]$^-$	355.3219	2.5	2
123	FA	FA(22:2)	[M-H]$^-$	335.2957	3.1	2
124	FA	FA(22:3)	[M-H]$^-$	333.28	2.7	2
125	FA	FA(22:4)	[M-H]$^-$	331.2643	2.5	2
126	FA	FA(22:5)	[M-H]$^-$	329.2487	2.3	2
127	FA	FA(22:6)	[M-H]$^-$	327.2331	2.2	2
128	FA	FA(23:0)	[M-H]$^-$	353.3426	4.9	2
129	FA	FA(23:0)	[M-H]$^-$	353.3427	4.6	2
130	FA	FA(23:0)	[M-H]$^-$	353.3427	5.0	2
131	FA	FA(23:1)	[M-H]$^-$	351.327	3.9	2
132	FA	FA(24:0)	[M-H]$^-$	365.3426	4.3	2
133	FA	FA(24:0-OH)	[M-H]$^-$	383.3532	3.4	3
134	FA	FA(24:1)	[M-H]$^-$	363.327	3.7	2
135	FA	FA(26:0)	[M-H]$^-$	395.3896	6.5	2
136	FA	FA(26:0-OH)	[M-H]$^-$	411.3845	4.0	3
137	FA	FA(26:1)	[M-H]$^-$	393.374	5.3	2
138	Ganglioside	asialo-GM1(d42:1)	[M+H]$^+$	1339.882	6.8	2
139	Ganglioside	asialo-GM1(d81:1/16:0)	[M+H]$^+$	1227.757	3.9	2
140	Ganglioside	GM3(d18:1/24:0)	[M-H]$^-$	1263.83	6.6	3
141	Hex2Cer	Hex2Cer(d18:1/24:0)	[M+H]$^+$	974.7494	6.9	2
142	Hex2Cer	Hex2Cer(d18:1/24:0-OH)	[M+H]$^+$	990.7451	6.8	2
143	Hex2Cer	Hex2Cer(d18:1/24:1)	[M+H]$^+$	972.7335	6.6	2
144	Hex2Cer	Hex2Cer(d34:1)	[M+CH_3COOH-H]$^-$	860.6106	4.5	2
145	Hex2Cer	Hex2Cer(d36:1)	[M+CH_3COOH-H]$^-$	888.6416	5.4	2
146	Hex2Cer	Hex2Cer(d42:1)	[M+CH_3COOH-H]$^-$	1032.755	6.8	2
147	Hex2Cer	Hex2Cer(t18:0/24:1)	[M+H]$^+$	990.7452	6.3	2
148	Hex3Cer	Hex3Cer(d18:1/24:0-OH)	[M+H]$^+$	1152.797	6.8	2

149	Hex3Cer	Hex3Cer(d18:1/26:0)	$[M+H]^+$	1164.834	7.1	2
150	Hex3Cer	Hex3Cer(d18:1/26:2-OH)	$[M+H]^+$	1180.829	7.0	2
151	Hex3Cer	Hex3Cer(t18:0/24:0)	$[M+H]^+$	1154.813	6.9	2
152	HexCer	HexCer(d18:0/24:0-OH)	$[M+H]^+$	830.7083	7.1	2
153	HexCer	HexCer(d18:0/30:1)	$[M+H]^+$	896.7919	7.7	2
154	HexCer	HexCer(d18:1/16:0)	$[M+H]^+$	700.5732	4.4	2
155	HexCer	HexCer(d18:1/23:0)	$[M+H]^+$	798.6825	6.9	2
156	HexCer	HexCer(d18:1/24:0)	$[M+H]^+$	812.6977	7.0	2
157	HexCer	HexCer(d18:1/24:0-OH)	$[M+H]^+$	828.6924	7.0	2
158	HexCer	HexCer(d18:1/24:1)	$[M+H]^+$	810.6821	6.7	2
159	HexCer	HexCer(d18:1/24:1)	$[M+H]^+$	810.6822	6.8	2
160	HexCer	HexCer(d18:1/25:0-OH)	$[M+H]^+$	842.7081	7.1	2
161	HexCer	HexCer(d18:1/26:0)	$[M+H]^+$	840.7287	7.4	2
162	HexCer	HexCer(d18:1/26:0-OH)_and_HexCer(d20:1/24:0-OH)	$[M+H]^+$	856.7235	7.3	2
163	HexCer	HexCer(d18:1/26:1-OH)_and_HexCer(d20:1/24:1-OH)	$[M+H]^+$	854.7078	6.8	2
164	HexCer	HexCer(d18:2/23:0)	$[M+H]^+$	796.6663	6.8	2
165	HexCer	HexCer(d32:1)	$[M+CH_3COOH-H]^-$	730.548	3.5	2
166	HexCer	HexCer(d32:1)	$[M+CH_3COOH-H]^-$	730.5482	4.0	2
167	HexCer	HexCer(d34:0)	$[M+CH_3COOH-H]^-$	760.5949	5.1	2
168	HexCer	HexCer(d34:1-OH)	$[M+CH_3COOH-H]^-$	714.553	4.5	2
169	HexCer	HexCer(d36:0)	$[M-H]^-$	788.582	5.7	2
170	HexCer	HexCer(d36:0)	$[M+CH_3COOH-H]^-$	788.6262	6.0	2
171	HexCer	HexCer(d36:0)	$[M+CH_3COOH-H]^-$	742.5841	5.4	2
172	HexCer	HexCer(d36:0-OH)	$[M+CH_3COOH-H]^-$	804.5767	5.3	2
173	HexCer	HexCer(d38:0)	$[M-H]^-$	802.6422	6.7	2
174	HexCer	HexCer(d38:0-OH)	$[M+CH_3COOH-H]^-$	832.6528	6.6	2

175	HexCer	HexCer(d38:1)	$[M-H]^-$	814.6421	6.5	2
176	HexCer	HexCer(d39:0)	$[M+CH_3COOH-H]^-$	830.6735	6.9	2
177	HexCer	HexCer(d40:0)	$[M+CH_3COOH-H]^-$	844.6886	6.9	2
178	HexCer	HexCer(d40:0-OH)	$[M+CH_3COOH-H]^-$	800.6624	6.7	2
179	HexCer	HexCer(d40:1	$[M+CH_3COOH-H]^-$	818.6287	6.9	2
180	HexCer	HexCer(d40:1-OH)	$[M+CH_3COOH-H]^-$	798.6468	6.8	2
181	HexCer	HexCer(d40:1-OH)	$[M+CH_3COOH-H]^-$	858.668	6.3	2
182	HexCer	HexCer(d41:0)	$[M+CH_3COOH-H]^-$	858.7043	7.1	2
183	HexCer	HexCer(d41:0-OH)	$[M+CH_3COOH-H]^-$	814.6783	7.0	2
184	HexCer	HexCer(d42:0)	$[M+CH_3COOH-H]^-$	872.72	7.2	2
185	HexCer	HexCer(d42:2-OH)	$[M+CH_3COOH-H]^-$	884.6836	6.8	2
186	HexCer	HexCer(d43:0)	$[M+CH_3COOH-H]^-$	886.7357	7.4	2
187	HexCer	HexCer(d43:1)	$[M+CH_3COOH-H]^-$	884.7203	7.3	2
188	HexCer	HexCer(d43:1-OH)	$[M+CH_3COOH-H]^-$	840.6934	7.2	2
189	HexCer	HexCer(d43:2-OH)	$[M-H]^-$	838.6781	6.9	2
190	HexCer	HexCer(d44:0)	$[M+CH_3COOH-H]^-$	900.7514	7.5	2
191	HexCer	HexCer(d44:0-OH)	$[M+CH_3COOH-H]^-$	856.7248	7.4	2
192	HexCer	HexCer(d44:0-OH)	$[M+CH_3COOH-H]^-$	916.7463	7.3	2
193	HexCer	HexCer(d44:1)	$[M+CH_3COOH-H]^-$	898.7359	7.2	2
194	HexCer	HexCer(d44:1-OH)	$[M+CH_3COOH-H]^-$	890.6858	7.0	2
195	HexCer	HexCer(t18:0/24:0-OH)	$[M+H]^+$	846.7027	6.8	2
196	HexCer	HexCer(t18:0/24:1-OH)	$[M+H]^+$	844.6872	6.5	2
197	HexCer	HexCer(t18:0/28:2)	$[M+H]^+$	884.7552	7.6	2
198	HexCer	HexCer(t18:0_22:0-OH)	$[M+H]^+$	818.6717	6.5	2

199	LPC	LPC(16:0)	$[M+CH_3COOH-H]^-$	554.3462	2.2	2
200	LPC	LPC(16:0/0:0)_and_ LPC(0:0/16:0)	$[M+H]^+$	496.34	2.1	2
201	LPC	LPC(18:0/0:0)_and_ LPC(0:0/18:0)	$[M+H]^+$	524.371	2.4	2
202	LPC	LPC(18:1)	$[M+H_2CO_2-H]^-$	506.3253	2.2	2
203	LPC	LPC(18:1/0:0)_and_ LPC(0:0/18:1)	$[M+H]^+$	522.3557	2.1	2
204	LPC	LPC(18:2)	$[M+CH_3COOH-H]^-$	578.3464	2.0	2
205	LPC	LPC(18:2/0:0)_and_ LPC(0:0/18:2)	$[M+H]^+$	520.34	1.8	2
206	LPC O-	LPC(O-16:0)	$[M+H]^+$	482.3606	2.2	3
207	LPE	LPE(0:0/18:1)	$[M-H]^-$	478.2941	2.2	3
208	LPE	LPE(18:0)	$[M-H]^-$	480.3096	2.5	2
209	LPE	LPE(18:0)	$[M-H]^-$	540.3315	2.1	2
210	LPE	LPE(18:0/0:0)	$[M+H]^+$	482.3242	2.4	2
211	LPE	LPE(18:1/0:0)	$[M-H]^-$	478.2941	2.3	2
212	LPE	LPE(18:2/0:0)	$[M-H]^-$	476.2785	2.0	3
213	LPE	LPE(20:0)	$[M-H]^-$	508.3409	2.5	2
214	LPE	LPE(20:0)	$[M-H]^-$	508.3409	2.4	2
215	LPE O-	LPE(O-18:0)	$[M-H]^-$	466.3304	2.3	2
216	LPE O-	LPE(O-18:1)	$[M-H]^-$	464.3147	2.3	3
217	PC	PC(14:0_16:0)	$[M+CH_3COOH-H]^-$	764.5453	4.7	2
218	PC	PC(16:0/16:0)	$[M+CH_3COOH-H]^-$	792.5766	5.7	2
219	PC	PC(16:0_16:1)	$[M+CH_3COOH-H]^-$	790.5608	4.9	2
220	PC	PC(16:0_18:1)	$[M+CH_3COOH-H]^-$	794.5482	5.8	2
221	PC	PC(16:0_18:3)	$[M+CH_3COOH-H]^-$	814.5609	4.3	2
222	PC	PC(16:0_20:1)_and_ PC(18:0_18:1)	$[M+H_2CO_2-H]^-$	772.5866	6.6	2
223	PC	PC(16:0_20:4)	$[M+CH_3COOH-H]^-$	840.5763	4.9	2
224	PC	PC(16:0_22:4)	$[M+CH_3COOH-H]^-$	868.6076	5.3	2
225	PC	PC(16:0_22:4)	$[M+CH_3COOH-H]^-$	868.6076	5.6	2

226	PC	PC(16:0_22:5)	[M+CH3COOH-H]-	866.592	5.0	2
227	PC	PC(16:0_22:6)	[M+CH3COOH-H]-	864.5766	4.7	2
228	PC	PC(17:0_18:2)	[M+CH3COOH-H]-	830.5922	5.5	2
229	PC	PC(17:0_18:2)	[M+CH3COOH-H]-	770.5708	6.0	2
230	PC	PC(18:0_20:3)	[M+CH3COOH-H]-	870.6233	6.3	2
231	PC	PC(18:0_20:4)_	[M+CH3COOH-H]-	868.6075	5.9	2
232	PC	PC(18:0_22:4)	[M+CH3COOH-H]-	896.639	6.5	2
233	PC	PC(18:0_22:6)	[M+CH3COOH-H]-	892.6078	5.7	2
234	PC	PC(18:1/18:1)	[M+CH3COOH-H]-	844.6078	6.0	2
235	PC	PC(18:1/18:1)	[M+CH3COOH-H]-	844.6075	5.8	2
236	PC	PC(18:1_18:2)	[M+CH3COOH-H]-	846.6235	6.6	2
237	PC	PC(18:1_20:4)	[M+CH3COOH-H]-	866.5922	5.3	2
238	PC	PC(18:2/18:2)	[M+CH3COOH-H]-	840.5764	4.5	2
239	PC	PC(31:0)	[M+H]+	720.5548	5.0	2
240	PC	PC(31:1)	[M+H]+	718.5387	5.4	2
241	PC	PC(32:0)	[M+H]+	734.57	5.5	2
242	PC	PC(33:0)	[M+H]+	748.5853	6.1	2
243	PC	PC(33:1)	[M+H]+	746.5707	5.1	2
244	PC	PC(33:2)	[M+H]+	744.5545	4.4	2
245	PC	PC(34:0)	[M+H]+	762.6013	6.5	2
246	PC	PC(34:1)	[M+H]+	760.5848	5.6	2
247	PC	PC(34:2)	[M+H]+	758.5695	4.9	2
249	PC	PC(35:1)	[M+H]+	774.601	6.1	2
250	PC	PC(35:2)	[M+H]+	772.5852	5.3	2
251	PC	PC(35:3)	[M+H]+	770.5701	5.9	2
252	PC	PC(36:1)	[M+H]+	788.6166	6.5	2
253	PC	PC(36:2)	[M+H]+	786.6005	5.8	2
254	PC	PC(36:2)	[M+H]+	786.6004	5.6	2
255	PC	PC(36:3)	[M+H]+	784.5846	4.9	2

256	PC	PC(36:4)	$[M+H]^+$	782.5694	4.7	2
257	PC	PC(36:4)	$[M+H]^+$	782.5699	4.8	2
258	PC	PC(37:1)	$[M+H]^+$	802.6324	6.8	2
259	PC	PC(38:2)	$[M+H]^+$	814.6324	6.5	2
260	PC	PC(38:2)	$[M+H]^+$	814.6324	6.8	2
261	PC	PC(38:3)	$[M+H]^+$	812.6163	6.1	2
262	PC	PC(38:5)	$[M+H]^+$	808.585	4.7	2
263	PC	PC(39:1)	$[M+H]^+$	830.6637	5.6	2
264	PC	PC(40:5)	$[M+H]^+$	836.6164	5.7	2
265	PC	PC(40:6)	$[M+H]^+$	834.6008	5.4	2
266	PC	PC(40:6)	$[M+H]^+$	834.6011	4.9	2
267	PC	PC(40:7)	$[M+H]^+$	832.5854	4.5	2
268	PC O-	PC(O-14:1/18:1)	$[M+CH_3COOH-H]^-$	774.5659	5.3	2
269	PC O-	PC(O-14:1/20:4)	$[M+CH_3COOH-H]^-$	796.554	5.7	2
270	PC O-	PC(O-15:1/20:4)	$[M+CH_3COOH-H]^-$	810.5661	5.4	2
271	PC O-	PC(O-16:0/14:0)	$[M+CH_3COOH-H]^-$	750.5659	5.4	2
272	PC O-	PC(O-16:0/18:2)	$[M+CH_3COOH-H]^-$	802.5973	5.7	2
273	PC O-	PC(O-16:0/22:5)	$[M+CH_3COOH-H]^-$	852.6129	6.3	2
274	PC O-	PC(O-16:1/18:0)	$[M+CH_3COOH-H]^-$	804.5918	6.8	2
275	PC O-	PC(O-16:1/18:1)	$[M+CH_3COOH-H]^-$	802.5972	6.3	2
276	PC O-	PC(O-16:1/18:2)	$[M+CH_3COOH-H]^-$	800.5814	5.5	2
277	PC O-	PC(O-16:1/20:3)	$[M+CH_3COOH-H]^-$	826.5971	5.5	2
278	PC O-	PC(O-17:0/18:2)	$[M+CH_3COOH-H]^-$	816.6137	6.6	2
279	PC O-	PC(O-17:0/18:2)	$[M+CH_3COOH-H]^-$	834.624	6.8	2
280	PC O-	PC(O-17:0/18:2)	$[M+CH_3COOH-H]^-$	816.6131	6.2	2
281	PC O-	PC(O-18:0/18:1)	$[M+H_2CO_2-H]^-$	816.613	6.4	2
282	PC O-	PC(O-18:0/18:2)	$[M+CH_3COOH-H]^-$	830.6293	6.6	2

283	PC O-	PC(O-18:0/20:3)	$[M+CH_3COOH-H]^-$	856.6438	6.6	2
284	PC O-	PC(O-18:0/20:3)	$[M+CH_3COOH-H]^-$	856.6442	6.7	2
285	PC O-	PC(O-18:0/20:4)	$[M+CH_3COOH-H]^-$	854.6283	6.5	2
286	PC O-	PC(O-18:0/20:4)	$[M+CH_3COOH-H]^-$	854.6284	6.2	2
287	PC O-	PC(O-18:1/18:0)	$[M+H_2CO_2-H]^-$	818.6288	6.7	2
288	PC O-	PC(O-18:1/18:1)	$[M+CH_3COOH-H]^-$	830.6287	6.4	2
289	PC O-	PC(O-18:1/18:1)	$[M+CH_3COOH-H]^-$	830.6287	6.8	2
290	PC O-	PC(O-18:1/18:2)	$[M+CH_3COOH-H]^-$	828.6125	5.7	2
291	PC O-	PC(O-18:1/18:2)	$[M+CH_3COOH-H]^-$	828.6131	6.5	2
292	PC O-	PC(O-18:1/18:2)	$[M+CH_3COOH-H]^-$	828.6129	6.0	2
293	PC O-	PC(O-18:1/20:3)	$[M+CH_3COOH-H]^-$	854.6283	6.0	2
294	PC O-	PC(O-18:1/20:4)	$[M+CH_3COOH-H]^-$	852.6126	5.6	2
295	PC O-	PC(O-18:1/22:4)	$[M+CH_3COOH-H]^-$	880.644	6.5	2
296	PC O-	PC(O-32:0)	$[M+H]^+$	720.5906	6.3	2
297	PC O-	PC(O-32:1)	$[M+H]^+$	718.5753	5.5	2
298	PC O-	PC(O-34:0)	$[M+H]^+$	748.6219	7.0	2
299	PC O-	PC(O-34:1)	$[M+H]^+$	746.6062	6.4	2
300	PC O-	PC(O-34:2)	$[M+H]^+$	744.5911	5.6	2
301	PC O-	PC(O-36:1)	$[M+H]^+$	774.6376	7.0	2
302	PC O-	PC(O-36:2)	$[M+H]^+$	772.622	6.5	2
303	PC O-	PC(O-36:4)	$[M+H]^+$	768.5883	6.4	2
304	PC O-	PC(O-16:1/20:4)	$[M+CH_3COOH-H]^-$	824.5816	5.4	2
305	PC O-	PC(O-38:5)	$[M+H]^+$	794.606	5.4	2
306	PC O-	PC(O-38:6)	$[M+H]^+$	792.5903	4.8	2
307	PC O-	PC(O-40:4)	$[M+H]^+$	824.6532	6.8	2
308	PE	PE(18:0_18:1)	$[M-H]^-$	744.5556	6.6	2
309	PE	PE(18:0_18:2)	$[M-H]^-$	742.5399	6.1	2
310	PE	PE(18:0_19:1)	$[M-H]^-$	758.5711	6.6	2
311	PE	PE(18:0_20:3)	$[M-H]^-$	768.5553	6.3	2

312	PE	PE(18:0_20:4)	[M-H]$^-$	766.5395	5.9	2
313	PE	PE(18:1/18:1)	[M-H]$^-$	742.5397	5.9	2
314	PE	PE(18:1_20:0)_and_PE(18:0_20:1)	[M-H]$^-$	772.5865	6.6	2
315	PE	PE(18:1_24:0)	[M-H]$^-$	828.6498	7.5	2
316	PE	PE(18:2_18:0)	[M+H]$^+$	744.5546	5.7	2
317	PE	PE(18:2_18:0)	[M+H]$^+$	744.5542	5.5	2
318	PE	PE(36:3)	[M+H]$^+$	742.5394	4.8	2
319	PE O-	PE(O-16:1/18:1)_and_PE(O-18:2/16:0)	[M+H]$^+$	702.5436	5.9	2
320	PE O-	PE(O-16:1/18:2)	[M-H]$^-$	698.5132	5.6	2
321	PE O-	PE(O-16:1/20:4)	[M-H]$^-$	722.5135	5.4	2
322	PE O-	PE(O-16:1/22:6)	[M+H]$^+$	748.5285	4.8	2
323	PE O-	PE(O-16:2/18:0)_and_PE(O-18:2/16:0)	[M-H]$^-$	700.5291	6.3	2
324	PE O-	PE(O-18:0/18:2)	[M-H]$^-$	714.5448	6.6	2
325	PE O-	PE(O-18:1/18:1)	[M+H]$^+$	730.5751	6.7	2
326	PE O-	PE(O-18:1/18:2)	[M-H]$^-$	726.5448	6.5	2
327	PE O-	PE(O-18:1/20:4)	[M-H]$^-$	750.5448	6.4	2
328	PE O-	PE(O-18:1/22:4)	[M-H]$^-$	778.5761	6.7	2
329	PE O-	PE(O-18:2/18:1)	[M-H]$^-$	726.5448	6.3	2
330	PE O-	PE(O-18:3/18:1)	[M-H]$^-$	728.5605	6.8	2
331	PG	PG(16:0_18:1)	[M-H]$^-$	747.5189	4.5	2
332	PG	PG(16:0_18:1)	[M-H]$^-$	747.5186	4.2	2
333	PG	PG(18:2_18:2)	[M-H]$^-$	769.5029	3.6	2
334	PG	PG(18:2_18:2)	[M-H]$^-$	769.503	3.4	2
335	PI	PI(16:0_18:2)	[M-H]$^-$	833.5191	3.9	2
336	PI	PI(16:0_20:4)	[M-H]$^-$	857.5189	3.8	2
337	PI	PI(18:0_18:2)	[M-H]$^-$	861.5499	4.6	2
338	PI	PI(18:0_20:3)	[M-H]$^-$	887.5659	4.9	2
339	PI	PI(18:0_20:4)	[M+H]$^+$	904.5908	4.5	2
340	PI	PI(18:0_22:6)	[M-H]$^-$	909.5502	4.4	2
341	PI	PI(18:1_18:2)	[M-H]$^-$	859.5346	3.9	2
342	PI	PI(18:1_20:4)	[M-H]$^-$	883.5351	3.9	2
343	PS	PS(18:0_18:1)	[M-H]$^-$	788.5451	5.4	2
344	PS	PS(36:1)	[M-H]$^-$	788.5452	4.2	3
345	PS	PS(36:2)	[M-H]$^-$	786.5295	4.8	3
346	PS	PS(38:4)	[M-H]$^-$	810.527	5.4	2

347	PS	PS(40:4)	$[M-H]^-$	898.5796	5.0	2
348	SM	SM(d32:0)	$[M+CH_3COOH-H]^-$	735.5665	4.1	2
349	SM	SM(d32:1)	$[M+H]^+$	675.5439	3.7	2
350	SM	SM(d32:1)	$[M+CH_3COOH-H]^-$	733.5505	4.2	2
351	SM	SM(d33:0)	$[M+CH_3COOH-H]^-$	749.5819	5.0	2
352	SM	SM(d33:1)	$[M+CH_3COOH-H]^-$	747.5664	4.2	2
353	SM	SM(d34:0)	$[M+H]^+$	705.5909	4.8	2
354	SM	SM(d34:0)	$[M+CH_3COOH-H]^-$	763.5974	5.0	2
355	SM	SM(d34:0-OH)	$[M+H]^+$	721.5857	4.4	2
356	SM	SM(d34:0-OH)	$[M+H]^+$	721.5858	4.0	2
357	SM	SM(d34:1)	$[M+H]^+$	703.5756	4.9	2
358	SM	SM(d34:2)	$[M+H]^+$	701.5597	3.8	2
359	SM	SM(d34:2)	$[M+CH_3COOH-H]^-$	759.5663	4.0	2
360	SM	SM(d35:0)	$[M+H]^+$	719.57	4.1	2
361	SM	SM(d35:0)	$[M+H]^+$	719.5703	3.4	2
362	SM	SM(d35:1)	$[M+H]^+$	717.5914	4.9	2
363	SM	SM(d35:1)	$[M+CH_3COOH-H]^-$	775.5976	5.1	2
364	SM	SM(d35:1)	$[M+CH_3COOH-H]^-$	775.5975	4.9	2
365	SM	SM(d36:0)	$[M+H]^+$	733.6225	5.8	2
366	SM	SM(d36:1)	$[M+H]^+$	731.6067	5.4	2
367	SM	SM(d36:1)	$[M+CH_3COOH-H]^-$	789.613	5.6	2
368	SM	SM(d36:2)	$[M+CH_3COOH-H]^-$	787.5976	4.8	2
369	SM	SM(d37:1)	$[M+CH_3COOH-H]^-$	803.6289	6.5	2
370	SM	SM(d38:0)	$[M+CH_3COOH-H]^-$	819.6602	6.7	2
371	SM	SM(d38:1)	$[M+H]^+$	759.6378	6.4	2
372	SM	SM(d40:0)	$[M+CH_3COOH-H]^-$	847.6914	7.1	2
373	SM	SM(d40:0-OH)	$[M+H]^+$	805.6797	6.7	2
374	SM	SM(d40:1)	$[M+CH_3COOH-H]^-$	845.6756	7.0	2
375	SM	SM(d40:1-OH)	$[M+H]^+$	803.6638	5.9	2

376	SM	SM(d40:2)	$[M+CH_3COOH-H]^-$	843.66	6.5	2
377	SM	SM(d40:2)	$[M+CH_3COOH-H]^-$	843.6605	6.6	2
378	SM	SM(d41:1)	$[M+H]^+$	801.685	7.3	2
379	SM	SM(d41:2)	$[M+CH_3COOH-H]^-$	857.6758	6.7	2
380	SM	SM(d42:0)	$[M+CH_3COOH-H]^-$	875.7223	7.5	2
381	SM	SM(d42:1)	$[M+H]^+$	815.7003	7.4	2
382	SM	SM(d42:2)	$[M+H]^+$	813.6843	6.9	2
383	SM	SM(d42:3)	$[M+H]^+$	811.6688	6.5	2
384	SM	SM(d43:0)	$[M+CH_3COOH-H]^-$	889.7381	7.7	2
385	SM	SM(d43:1)	$[M+H]^+$	829.7161	7.8	2
386	SM	SM(d44:0)	$[M+CH_3COOH-H]^-$	903.7536	8.0	2
387	SM	SM(d44:1)	$[M+H]^+$	843.7311	8.0	2
388	SM	SM(d44:2)	$[M+CH_3COOH-H]^-$	899.7229	7.3	2
389	SM	SM(t34:0)_or_SM(d34:0-OH)	$[M+CH_3COOH-H]^-$	779.5923	4.6	2
390	SM	SM(t34:0)_or_SM(d34:0-OH)	$[M+CH_3COOH-H]^-$	779.5924	4.2	2
391	SM	SM(t39:0)_or_SM(d39:0-OH)	$[M+CH_3COOH-H]^-$	831.6602	6.8	2
392	SM	SM(t40:0)_or_SM(d40:0-OH)	$[M+CH_3COOH-H]^-$	863.6864	6.7	2
393	SM	SM(t42:1)_or_SM(d42:1-OH)	$[M+H]^+$	831.6952	6.7	2
394	SM	SM(t42:1)_or_SM(d42:1-OH)	$[M+CH_3COOH-H]^-$	889.702	6.7	2
395	TG	TG(10:0_16:0_18:1)	$[M+NH_4]^+$	766.6923	8.0	2
396	TG	TG(12:0_16:0_18:1)_and_TG(10:0_18:0_18:1)_and_TG(14:0_16:0_16:1)	$[M+NH_4]^+$	794.7235	8.4	2
397	TG	TG(14:0_16:0_16:0)_and_TG(12:0_16:0_18:0)	$[M+NH_4]^+$	796.7398	8.8	2
398	TG	TG(14:0_16:0_18:2)_and_TG(14:0_16:1_18:1)	$[M+NH_4]^+$	820.7396	8.4	2

399	TG	TG(15:0_16:0_18:0)_and_TG(16:0_16:0_17:1)_and_TG(16:0_16:1_17:0)	$[M+NH_4]^+$	836.77	9.0	2
400	TG	TG(15:0_18:1_18:1)_and_TG(16:0_17:1_18:1)_and_TG(16:1_17:0_18:1)	$[M+NH_4]^+$	862.7857	9.0	2
401	TG	TG(15:0_18:1_18:2)_and_TG(16:0_17:1_18:2)_and_TG(16:1_17:1_18:1)	$[M+NH_4]^+$	860.7703	8.6	2
402	TG	TG(15:0_18:1_18:2)_and_TG(16:0_17:1_18:2)_and_TG(16:1_17:1_18:1)	$[M+NH_4]^+$	860.7704	8.5	2
403	TG	TG(16:0_16:0_16:1)_and_TG(14:0_16:0_18:1)	$[M+NH_4]^+$	822.7556	8.8	2
404	TG	TG(16:0_16:0_17:0)_and_TG(15:0_16:0_18:0)_and_TG(14:0_16:0_19:0)	$[M+NH_4]^+$	838.786	9.4	2
405	TG	TG(16:0_16:0_17:0)_and_TG(15:0_16:0_18:0)_and_TG(14:0_16:0_19:0)	$[M+NH_4]^+$	838.7864	9.3	2
406	TG	TG(16:0_16:0_20:0)	$[M+NH_4]^+$	880.8323	9.7	2
407	TG	TG(16:0_16:1_18:1)_and_TG(14:0_18:1_18:1)	$[M+NH_4]^+$	848.7695	8.8	2
408	TG	TG(16:0_18:1_18:2)	$[M+NH_4]^+$	874.7857	8.8	2
409	TG	TG(16:0_18:1_22:4)	$[M+NH_4]^+$	926.8165	8.4	2
410	TG	TG(16:0_18:1_22:4)	$[M+NH_4]^+$	926.8174	9.1	2
411	TG	TG(16:0_18:1_22:6)	$[M+NH_4]^+$	922.7857	8.5	2
412	TG	TG(16:0_18:1_24:0)_and_TG(16:0_16:0_26:1)_and_TG(18:0_18:1_22:0)	$[M+NH_4]^+$	962.9111	10.0	2
413	TG	TG(16:0_18:2_18:3)	$[M+NH_4]^+$	870.7547	8.2	2
414	TG	TG(16:0_18:2_20:4)	$[M+NH_4]^+$	896.7704	8.3	2
415	TG	TG(16:0_18:2_22:6)	$[M+NH_4]^+$	920.7699	8.2	2
416	TG	TG(16:1_18:1_18:2)	$[M+NH_4]^+$	872.77	8.5	2

417	TG	TG(17:0_18:1_18:2)_and_TG(17:1_18:1_18:1)_and_TG(16:1_18:1_19:1)_and_TG(16:0_18:1_19:2)	$[M+NH_4]^+$	888.8011	9.0	2
418	TG	TG(17:0_18:2_18:2)_and_TG(17:1_18:1_18:2)	$[M+NH_4]^+$	886.7861	8.7	2
419	TG	TG(17:1_18:2_18:2)	$[M+NH_4]^+$	884.7704	8.3	2
420	TG	TG(18:0_16:0_22:6)	$[M+NH_4]^+$	924.8019	9.0	2
421	TG	TG(18:0_18:0_18:1)_and_TG(16:1_18:0_20:0)_and_TG(16:0_18:0_20:1)	$[M+NH_4]^+$	906.8481	9.6	2
422	TG	TG(18:0_18:2_22:6)	$[M+NH_4]^+$	948.8021	8.6	2
423	TG	TG(18:1_18:1_18:2)	$[M+NH_4]^+$	900.8013	8.8	2
424	TG	TG(18:1_18:1_20:1)_and_TG(18:1_18:2_20:0)_and_TG(18:0_18:2_20:1)_and_TG(16:0_18:1_22:2)	$[M+NH_4]^+$	930.8481	9.5	2
425	TG	TG(18:1_18:1_20:4)_and_TG(16:0_18:1_22:5)_and_TG(18:1_18:2_20:3)	$[M+NH_4]^+$	924.8015	8.8	2
426	TG	TG(18:1_18:2_18:2)	$[M+NH_4]^+$	898.7856	8.4	2
427	TG	TG(18:1_18:2_19:1)_and_TG(18:2_18:2_19:0)_and_TG(18:1_18:1_19:2)	$[M+NH_4]^+$	914.8175	9.1	2
428	TG	TG(18:1_18:2_19:1)_and_TG(18:2_18:2_19:0)_and_TG(18:1_18:1_19:2)	$[M+NH_4]^+$	914.8174	9.3	2
429	TG	TG(18:1_18:2_20:1)_and_TG(18:1_18:1_20:2)	$[M+NH_4]^+$	928.8324	9.3	2
430	TG	TG(18:1_18:2_20:1)_and_TG(18:1_18:1_20:2)	$[M+NH_4]^+$	928.8324	9.4	2
431	TG	TG(18:2/18:2/18:2)	$[M+NH_4]^+$	896.77	8.1	2
432	TG	TG(48:3)	$[M+NH_4]^+$	818.7231	8.0	2
433	TG	TG(49:3)	$[M+NH_4]^+$	832.7387	8.2	2
434	TG	TG(50:3)	$[M+NH_4]^+$	846.7533	8.4	2

435	TG	TG(51:2)	$[M+NH_4]^+$	862.7856	9.1	2
436	TG	TG(52:6)	$[M+NH_4]^+$	868.7386	7.9	2
437	TG	TG(53:1)	$[M+NH_4]^+$	892.8329	9.0	2
438	TG	TG(53:2)	$[M+NH_4]^+$	890.8169	9.4	2
439	TG	TG(54:7)	$[M+NH_4]^+$	894.7549	7.8	2
440	TG	TG(54:7)	$[M+NH_4]^+$	894.7545	7.9	2
441	TG	TG(55:2)	$[M+NH_4]^+$	918.8485	9.5	2
442	TG	TG(56:1)	$[M+NH_4]^+$	934.8798	9.8	2
443	TG	TG(56:2)	$[M+NH_4]^+$	932.8641	9.6	2
444	TG	TG(56:7)	$[M+NH_4]^+$	922.7863	8.3	2
445	TG	TG(56:8)	$[M+NH_4]^+$	920.7702	8.0	2
446	TG	TG(58:10)	$[M+NH_4]^+$	944.7705	7.8	2
447	TG	TG(58:2)	$[M+NH_4]^+$	960.8953	9.8	2
448	TG	TG(58:3)	$[M+NH_4]^+$	958.88	9.6	2
449	TG	TG(58:4)	$[M+NH_4]^+$	956.8642	9.5	2
450	TG	TG(58:5)	$[M+NH_4]^+$	954.8486	9.4	2
451	TG	TG(58:6)	$[M+NH_4]^+$	952.8328	9.1	2
452	TG	TG(58:9)	$[M+NH_4]^+$	946.7857	8.2	2
453	TG	TG(60:3)	$[M+NH_4]^+$	986.9108	9.8	2
